# Poly(sodium styrene sulfonate)-Grafted SiO_2_ Nanoparticle: Synthesis and Use as a Water-Insoluble Dispersant for Coal Water Slurry

**DOI:** 10.3390/polym17010021

**Published:** 2024-12-25

**Authors:** Guanghua Zhang, Ruijun Liu, Wanbin Zhang, Kangmin Zhang, Junfeng Zhu, Ce Zhang

**Affiliations:** 1 Shaanxi Collaborative Innovation Center of Industrial Auxiliary Chemistry and Technology, Shaanxi University of Science and Technology, Xi’an 710021, China; zhangce@sust.edu.cn; 2 Shaanxi Key Laboratory of Chemical Additives for Industry, College of Chemistry and Chemical Engineering, Shaanxi University of Science and Technology, Xi’an 710021, China; lrj1820593101@163.com (R.L.); 220812083@sust.edu.cn (K.Z.); zhujunfeng@sust.edu.cn (J.Z.); 3 Key Laboratory of Auxiliary Chemistry and Technology for Chemical Industry, Ministry of Education, Shaanxi University of Science and Technology, Xi’an 710021, China

**Keywords:** coal water slurry, silicon dioxide, PSSNa, SI-ATRP, graft polymer

## Abstract

This study introduces a novel water-insoluble dispersant for coal water slurry (CWS), namely, a poly(sodium styrene sulfonate)-*grafted* SiO_2_ nanoparticle (SiO_2_-*g*-PSSNa). SiO_2_-*g*-PSSNa was synthesized by combining the surface acylation reaction with surface-initiated atom transfer radical polymerization (SI-ATRP). Fourier transform infrared spectrometry (FTIR), X-ray photoelectron spectroscopy (XPS), energy dispersive spectrometer (EDS), nuclear magnetic resonance spectroscopy (NMR) and thermogravimetric analysis (TGA) verified that SiO_2_-*g*-PSSNa with the desired structure was successfully obtained. Afterwards, the performance of SiO_2_-*g*-PSSNa as a dispersant in CWS preparation was evaluated. The results indicated that the optimal dosage of SiO_2_-*g*-PSSNa was 0.3%. Compared to the famous commercial products, PSSNa and lignosulfonate (LS), SiO_2_-*g*-PSSNa exhibits improved viscosity reduction performance. When SiO_2_-*g*-PSSNa was used as the dispersant, the maximum coal loading of CWS was 64.2%, which was higher than LS (63.4%) and PSSNa (63.9%). All CWSs obtained in this study were pseudoplastic fluids and more consistent with the Herschel–Bulkley rheological model. The turbiscan stability index (TSI) of CWS prepared with SiO_2_-*g*-PSSNa was 0.05, which was significantly lower than CWSs obtained from PSSNa (0.30) and LS (0.36). Therefore, SiO_2_-*g*-PSSNa also exhibits excellent stability performance. This result was confirmed by rod penetration tests. The underlying mechanism was also clarified by various measurements, such as contact angle, zeta potential, EDS and low-field nuclear magnetic resonance spectra (low-field NMR). The results reveal that SiO_2_-*g*-PSSNa can adsorbed onto the coal surface. SiO_2_-*g*-PSSNa possesses a special branched structure, which bears a higher charge density as compared to linear ones with approximate chemical composition. As a result, coal particles adsorbed with SiO_2_-*g*-PSSNa exhibit more electronegativity. With the enhancement of the electrostatic repulsive between coal particles, the apparent viscosity was lowered and the static stability was improved. This study demonstrated that solubility in water is not an essential factor in engineering the dispersant. Densely charged groups are probably more important.

## 1. Introduction

The use of coal in an efficient and low-polluting manner is of great significance to the world [1]. During recent decades, coal water slurry (CWS) has attracted constant attention due to its advantages, such as low pollution, low cost, and high efficiency [2,3,4,5,6]. CWS is recognized as a clean coal-based fluidized fuel and an important raw material for the production of chemicals [7,8,9]. It is generally composed of pulverized coal particles with different sizes, water, and dispersants in a small proportion [10,11,12]. Due to CWS being a thermodynamically unstable system, the coal particles are prone to aggregate due to the driving force of hydrophobic interaction [13,14]. This causes a rise in apparent viscosity and a degradation of stability. Obviously, this is unfavorable for the transport, pumping, and spraying of CWS. Thus, dispersants play a critical role in the CWS system, although its proportion is very small [15,16]. Generally speaking, dispersants can absorb onto the coal surface and endow coal particles with enhanced electronegativity and steric hindrance, which will suppress the aggregation behavior of coal particles. As a result, the apparent viscosity of CWS is lowered and the stability is improved.

Over the last decades, considerable efforts have been devoted to developing novel dispersants with excellent dispersion and stability performance. One of the categories is natural macromolecular dispersants, including lignin [17,18], humic acid (HA), tannic acid (TA) [19,20], cellulose [21], and their derivatives [22]. Among these dispersants, lignosulfonate (LS) is one of the most famous commercially available products. Recently, HA received growing attention since it possesses analogous backbone structure to coal. Zhang reported the synthesis and performance of three amphiphilic HA-based polymer dispersants [23]. Our group developed a HA-*g*-poly (sodium styrene sulfonate) (HA-*g*-PSSNa) dispersant, and its dispersion and stability performance were superior to the PSSNa dispersant without an HA backbone [24]. Natural CWS dispersants have advantages, such as cost-effectiveness, wide availability, and environmental friendliness. However, their dispersion performance is usually limited and unsuitable for the preparation of CWS using low-rank coal. Synthetic polymer dispersants generally exhibit improved dispersion performance, which has been widely studied and used recently. Naphthalene sulfonic formaldehyde condensate (NSF) and sulphonated acetone–formaldehyde (SAF) are the most widely used market products [25,26]. In recent years, research has mainly focused on the synthesis and performance of polycarboxylic acid (PC) and PSSNa dispersants [27,28]. For instance, Zhu reported the synthesis of PC dispersant through reversible addition–fragmentation chain transfer polymerization (RAFT), and the dispersion mechanism was explained at a molecular level [29,30]. Zhang synthesized a series of PSSNa dispersants with controllable topological architecture [31]. Overall, in recent decades, plenty of CWS dispersants with different structures have been reported. As far as we know, nearly all of these dispersants are water-soluble polymers, which bear a large number of anionic groups. Water-insoluble dispersants have rarely been reported.

Recently, besides developing new types of dispersants, a novel strategy to improve the rheological behavior of CWS has been proposed, that is, introducing a second liquid or a second particle into the CWS system. Liu’s study demonstrated that adding the second fluid (such as diesel, solvent oil, etc.) and the second particle (such as glass bead, Teflon, etc.) can significantly lower the apparent viscosity of CWS [32]. Inspired by Liu’s study, we incorporated polystyrene (PS) microspheres into the CWS and found that PS microspheres can enhance the adsorption of the NSF dispersant. Accordingly, the apparent viscosity of CWS was lowered. Nevertheless, although this strategy is encouraging, it still cannot avoid using dispersants. The preparation of CWS with desired performance by adding water-insoluble particles instead of conventional water-soluble polymer dispersants is a challenging and interesting topic.

Therefore, in this study, we attempted to clarify whether water-insoluble particles with a large number of charged groups can be used as dispersants for CWS. A novel water-insoluble dispersant composed of SiO_2_ core and PSSNa side segments was synthesized, named poly(sodium styrene sulfonate)-*grafted*-SiO_2_ (SiO_2_-*g*-PSSNa). SiO_2_ nanoparticle was used as a precursor. PSSNa segments were grafted onto its surface by combining the surface acylation reaction with surface-initiated atom transfer radical polymerization (SI-ATRP) [33] after a detailed structural characterization. The performance of SiO_2_-*g*-PSSNa used as a dispersant in CWS preparation was systematically evaluated by measuring apparent viscosity, static stability, contact angles, zeta potential, etc. Finally, by comparing with the other two commercial water-soluble dispersants, PSSNa and LS, the dispersing and stabilizing mechanism of SiO_2_-*g*-PSSNa was proposed.

## 2. Experimental Section

### 2.1. Materials

Functionalized nano silica (SiO_2_-NH_2_, 20 nm, 99 wt.%) for NH_2_ groups was purchased from Jiangsu Xianfeng Nano Material Technology Co., Ltd., Nanjing, China, and synthesized by the surface modification reaction between SiO_2_ nanoparticles and KH550. The SEM and particle size distribution are shown in Figure S1. Dichloromethane (99.9%) was obtained from Shanghai Titan Scientific Co., Ltd., Shanghai, China. 2-Bromo-2-methylpropionyl bromide (BIBB, 98%) and 4-vinyl benzene sulfonic acid sodium salt hydrate (SSNa, 90%) were purchased from Adamas and used as received. 2,2′-Bipyridine (Bpy, 99%) was purchased from Aladdin. Cu(I)Cl (98%) was purchased from Adamas. Before use, Cu(I)Cl was treated by stirring with acetic acid until a white color was observed, before being filtered and washed with ethanol. After drying under vacuum at room temperature, pure Cu(I)Cl was obtained and stored in a sealed atmosphere. Triethylamine (TEA, ≥99%) was obtained from Tianjin Tianli Chemical Reagent Co., Ltd., Tianjin, China.

### 2.2. Synthesis of Poly(sodium styrene sulfonate)-Grafted SiO_2_ Nanoparticle (SiO_2_-g-PSSNa)

SiO_2_-*g*-PSSNa was synthesized via SI-ATRP. Before polymerization, SI-ATRP initiator SiO_2_-Br was synthesized by the surface acylation reaction between SiO_2_-NH_2_ and BIBB in the presence of TEA. Briefly, SiO_2_-NH_2_ (1.2 g) and TEA (4.48 mL, 32.36 mmol) were dissolved in 30 mL of anhydrous CH_2_Cl_2_ thoroughly. Then, BIBB (4 mL, 32.36 mmol) was added dropwise into the suspension within 30 min at around 0 °C. After the addition, the mixture was allowed to react for another 11.5 h at room temperature. Finally, the suspension was centrifuged at 8000 rpm for 5 min. In order to remove the unreacted BIBB and salt completely, the crude product was washed with deionized water until neutral, and then dialyzed in water (molecular weight cut off: 500) for at least 24 h. After drying at 85 °C for 48 h, the SiO_2_-Br was obtained as a white solid. Yield: 83%.

In the following step, SiO_2_-Br obtained above was used as the initiator. The synthetic procedure for SiO_2_-*g*-PSSNa is described as follows. Firstly, 0.2 g SiO_2_-Br was homogeneously dispersed into water under ultrasound. Then, Bpy (0.15 g, 0.96 mmol) and SSNa (6 g, 29 mmol) were added under stirring. The mixture was bubbled with N_2_ for 20 min, and then Cu(I)Cl (0.048 g, 0.48 mmol) was added quickly under the N_2_ atmosphere. After bubbling with N_2_ for another 20 min, the mixture was sealed off and heated to 90 °C. The polymerization was carried out at this temperature for 12 h. After polymerization, the unreacted SSNa monomer, copper salt, and ligand were removed by dialyzing against distilled water and ethanol sequentially. The final product of SiO_2_-*g*-PSSNa was obtained after drying at 85 °C for 72 h.

### 2.3. Preparation of CWS Using SiO_2_-g-PSSNa as a Dispersant

In this study, CWSs were prepared using Shenhua coal. The approximate and ultimate analyses of coal were carried out according to GB/T 31391-2015 [34] and GB/T 212-2008 [35], and the results are shown in Table S1. In order to improve the packing density of CWSs, a bi-modal gradation technology described in the literature was employed, and the size distribution of blended coal is shown in Figure S3 [31]. The preparation of CWS included the following steps. Firstly, 80 g blended coal was weighed and stirred evenly. The required quantity of water and SiO_2_*-g*-PSSNa was calculated according to the preset coal loading of CWS and the dosage of dispersant, respectively. SiO_2_*-g*-PSSNa was uniformly dispersed into water under ultrasound. Then, blended coal was slowly added to the SiO_2_*-g*-PSSNa suspension under stirring. After stirring for 10 min, CWS was left to stand for another 10 min and subject to various tests.

### 2.4. Measurements

#### 2.4.1. Morphological Characterization of SiO_2_-*g*-PSSNa

The morphology of SiO_2_-*g*-PSSNa was determined using an SU8100 scanning electron microscope (SEM, Hitachi, Tokyo, Japan) and transmission electron microscopy (TEM, FEI, Tecnai G2 F20 S-TWIN, Hillsboro, OR, USA).

#### 2.4.2. Fourier Transform Infrared Spectrometry (FTIR)

FTIR spectra of the products were tested on a VECTOR-22 (Bruker Instruments, Karlsruhe, Germany) instrument with wavenumbers ranging from 4000 cm^−1^ to 400 cm^−1^. The dried sample and KBr were mixed thoroughly at the concentration of 1 to 100 mg in a mortar. Then, the mixture was pressed into a disc for measurement.

#### 2.4.3. X-Ray Photoelectron Spectroscopy (XPS)

XPS were recorded on a Kratos AXIS SUPRA spectrometer (Kratos, Manchester, UK) using a monochromatic Al Kα source operated at 150 W (10 mA, 15 KV).

#### 2.4.4. Nuclear Magnetic Resonance Spectroscopy (NMR)

^1^H NMR spectrum and ^13^C NMR spectrum were recorded on a Bruker Avance II 600 MHz NMR spectrometer (Bruker Instruments, Karlsruhe, Germany), with D_2_O as the solvent.

#### 2.4.5. Thermogravimetric Analysis (TGA)

TGA measurements were conducted on a Q600 SDT instrument (TA, New Castle, DE, USA). The temperature ranged from room temperature to 800 °C with a heating rate of 20 °C/min under a nitrogen atmosphere.

#### 2.4.6. Energy Dispersive Spectrometer (EDS)

The EDS was recorded on an S8100 scanning electron microscope (SEM), which was equipped with an EDS unit. Samples were scanned directly without spraying gold.

#### 2.4.7. Evaluation of the Apparent Viscosity and Rheological Behavior of CWSs

The apparent viscosity as well as the rheological behavior of CWSs were measured on an LC-HBT-1 viscometer (Lichen Instrument Factory, Shanghai, China). The viscosity at the shear rates of 10, 20, 40, 60, 80, and 100 s^−1^ were respectively recorded. The apparent viscosity of CWSs was determined as the average of ten measurements at 100 s^−1^.

The dependence of shear stress versus shear rate was fitted using the Herschel–Bulkley Equation (1) and the power-law Equation (2) model, as described below [36,37].

(1) $$\tau =\tau_{0}+k\gamma^{n}$$

(2) $$\tau =k\gamma^{n}$$

where *τ* is the shear stress (Pa), *τ*_0_ is the yield stress (Pa), k is the consistency coefficient (Pa·s), η is the plastic viscosity (Pa·s), *γ* is the shear rate (s^−1^), and n is the rheological index.

#### 2.4.8. The Static Stability of CWSs

The static stability of CWSs was evaluated using rod penetration tests and a measure of the turbiscan stability index (TSI) [38,39]. In rod penetration tests, the penetration rate was calculated according to Equation (3):

(3) $$Penetration\;ratio\left(\%\right)=d/d_{t}\times 100\%$$

where *d* is the falling distance (mm), and *d_t_* is the maximum falling distance (mm).

TSI values of CWSs were monitored on a Turbiscan LAB stability analyser (Formulaction, Toulouse, France). Scans were recorded every 10 min for 3 h.

#### 2.4.9. Measurements of the Contact Angles

The contact angle between the dispersant solutions (or suspension) and the coal was measured using a DSA100 dynamic contact angle measuring instrument (Kruss, Heidelberg, Germany). The coal blocks were polished with sandpaper until the surface was smooth. The distilled water, SiO_2_-*g*-PSSNa suspension, and PSSNa and LS solutions were dropped onto the surface of the coal lump. Each sample was tested 5 times, and the standard deviation was calculated. The concentration of the dispersant solutions (or suspension) was approximate to that in CWS, with 63.5% coal loading and 0.3% dispersant dosage.

#### 2.4.10. Measurements of the Zeta Potential on the Coal Particle Surface

The zeta potential of coal particles adsorbed with dispersants was determined on a Nano-ZS90 DLS (Malvern Instruments, Malvern, UK). Then, 0.2 g blended coal was dispersed into 30 mL deionized water or dispersant solutions. The concentration of the dispersant solutions (or suspension) was the same as those in contact angle measurements. The mixture was shaken in a 25 °C water bath for 5 h and centrifuged at 8000 rpm for 10 min; then, the supernatant was subject to zeta potential measurements. Each sample was measured 5 times, and the standard deviation was calculated.

#### 2.4.11. Characterization of Low-Field NMR

Low-field NMR characterizations were carried out on a MesoMR23-060H-I analyzer (Niumag, Soochow, China). CWS samples prepared using LS and SiO_2_-*g*-PSSNa as the dispersants were subjected to measurements, respectively.

## 3. Results and Discussion

### 3.1. Synthesis and Characterization of SiO_2_-g-PSSNa

**Scheme 1:**
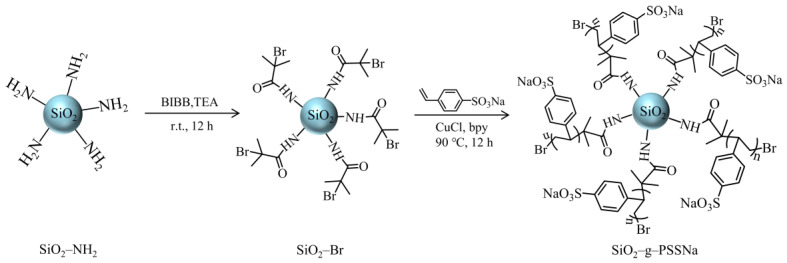
Synthetic route of SiO_2_-*g*-PSSNa.

**Figure 1:**
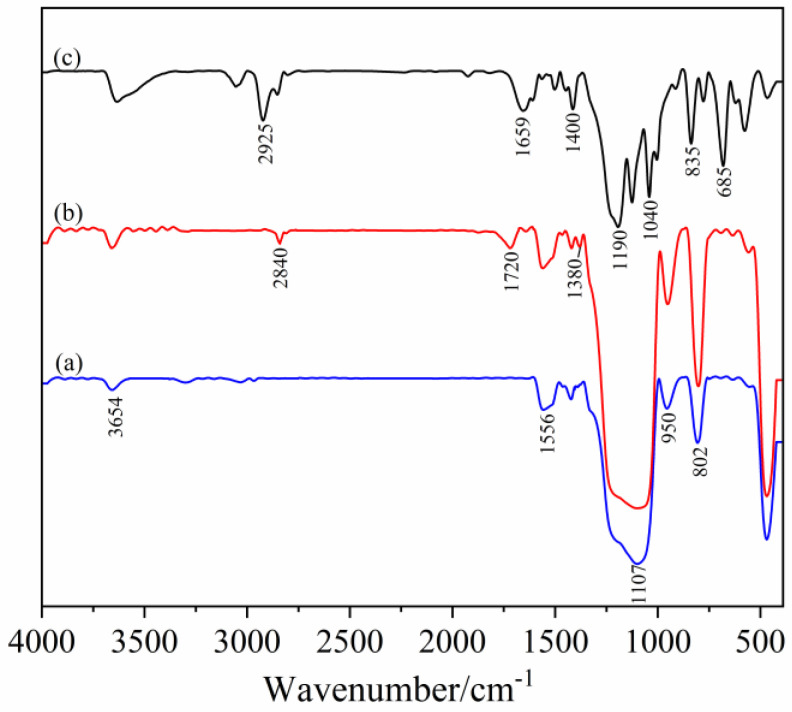
FTIR spectra of (**a**) SiO_2_-NH_2_, (**b**) SiO_2_-Br, and (**c**) SiO_2_-*g*-PSSNa.

**Figure 2:**
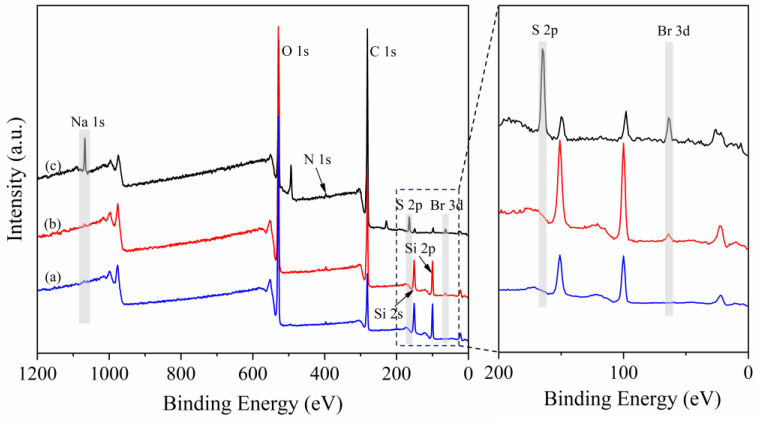
XPS spectra of (**a**) SiO_2_-NH_2_, (**b**) SiO_2_-Br, and (**c**) SiO_2_-*g*-PSSNa.

The synthesis of SiO_2_-*g*-PSSNa involves two main steps, as described in Scheme 1. Firstly, a SiO_2_ nanoparticle bearing SI-ATRP initiation points (SiO_2_-Br) was synthesized via a surface acylation reaction. Figure S2 shows the SEM and TEM images of SiO_2_-*g*-PSSNa, from which it can be seen that after grafting, the PSSNa segments are entangled with each other, the SiO_2_ particles are coated by PSSNa chains, and the particles are agglomerated with each other. Figure 1a,b display the FTIR spectra of SiO_2_-NH_2_ and SiO_2_-Br, respectively. In comparison to Figure 1a,b, the strong absorption peak at around 1107 cm^−1^ was retained, indicating that the Si-O-Si bonds were not broken during the acylation reaction. In Figure 1b, the absorption peaks at 1720 cm^−1^ and 1380 cm^−1^, respectively, attributed to the vibration of C=O and –CH_3_ groups can be detected clearly, which indicates that an acylation reaction was conducted. XPS and EDS measurements provided further evidence for successfully obtaining SiO_2_-Br, as shown in Figure 2b and Table 1. Apparently, after the reaction, the Br element was incorporated.

**Figure 3:**
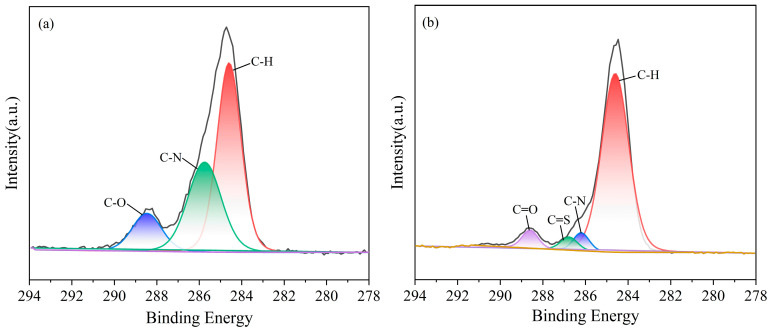
C 1s spectra of (**a**) SiO_2_-NH_2_ and (**b**) SiO_2_-*g*-PSSNa.

**Figure 4:**
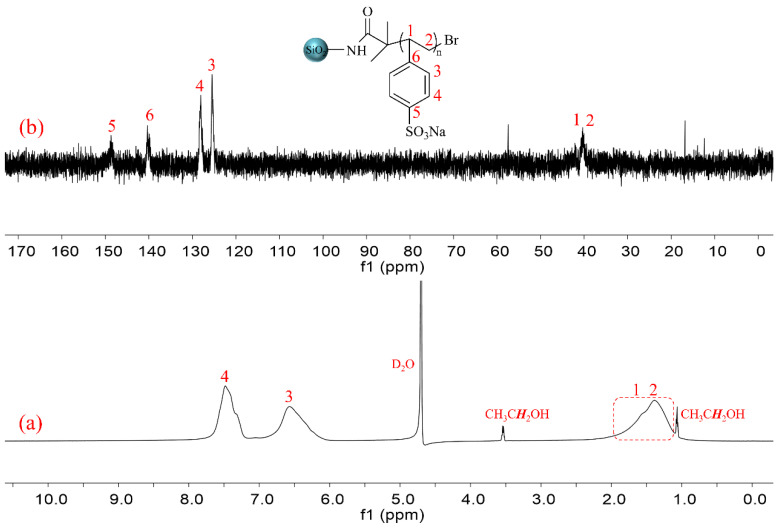
Typical (**a**) ^1^H NMR spectrum and (**b**) ^13^C NMR spectrum of SiO_2_-*g*-PSSNa.

In the next step, the previously obtained SiO_2_-Br was used as the initiator, and SI-ATRP was employed for the synthesis of SiO_2_-*g*-PSSNa. Figure S4 shows that after the introduction of PSSNa segments, the aqueous solution of SiO_2_ nanoparticles exhibits improved stability. Figure 2c shows the FTIR spectrum of SiO_2_-*g*-PSSNa, where the absorption at 1190 cm^−1^ and 1040 cm^−1^ was caused by the antisymmetric and symmetrical vibrational absorption of -SO_3_Na groups [40,41]. Meanwhile, the characteristic band of the benzene ring at around 650~900 cm^−1^ can also be clearly detected. XPS and EDS give more structural information on SiO_2_-*g*-PSSNa, as shown in Figure 2c and Table 1. The elemental composition determined by both XPS and EDS indicates that after polymerization, Na and S elements were incorporated. Figure 3 shows the C 1s spectra of SiO_2_-NH_2_ and SiO_2_-*g*-PSSNa. As compared to Figure 3a, the detection of binding energy attributed to the C-SO_3_^−^Na^+^ bond at 287.1 eV in Figure 3b further confirmed that PSSNa segments were incorporated [42]. Figure 4 shows the typical 1H NMR spectrum (a) and 13C NMR spectrum (b) of SiO_2_-*g*-PSSNa. In Figure 4a, the chemical shift of δ = 1.45 ppm is attributed to the methyl and methylene protons in the backbone, and the chemical shifts at δ = 7.54 and 6.66 ppm are caused by protons in the benzene ring. In Figure 4b, the carbon attributed to methylene (δ = 40.1 ppm) and benzene ring (δ = 148.7 ppm) can be clearly detected. In any case, the chemical shift of the double-bonded proton at δ = 5.25, 5.80 ppm is absent, indicating that the unreacted monomer was thoroughly removed and the purified polymer dispersant was obtained.

In addition, the zeta potentials of SiO_2_-NH_2_ and SiO_2_-*g*-PSSNa particles were compared, as listed in Table 2. Table 2 implies that SiO_2_-NH_2_ nanoparticles exhibit a weak positive charge. This phenomenon is caused by the presence of NH_2_ groups. After polymerization, the surface potential of particles became strongly negative. This is reasonable as PSSNa segments bear abundant -SO_3_Na negatively charged groups. Therefore, all the above analyses verified that SiO_2_-*g*-PSSNa was successfully synthesized.

### 3.2. The Thermal Decomposition Behavior of SiO_2_-g-PSSNa

**Figure 5:**
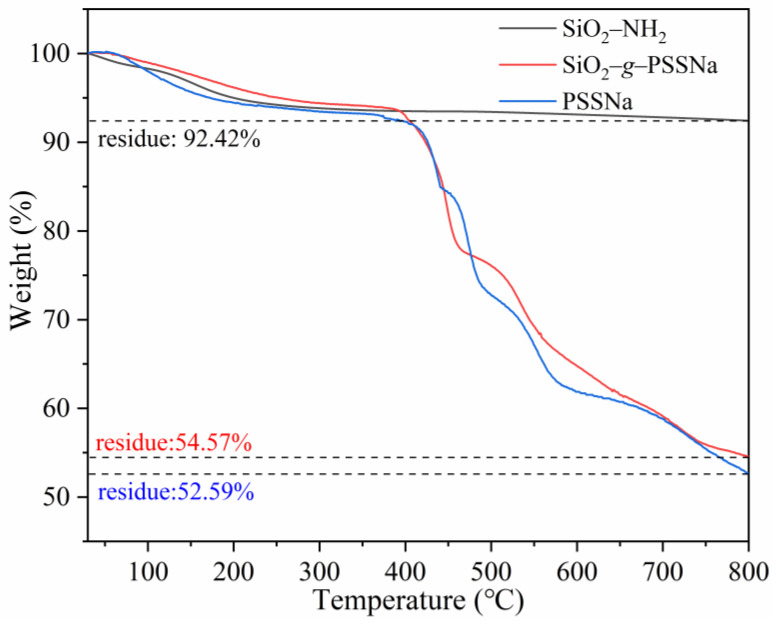
The TG curves of SiO_2_-NH_2_, PSSNa, and SiO_2_-*g*-PSSNa nanoparticles.

The thermal decomposition behavior of SiO_2_-NH_2_, PSSNa and SiO_2_-*g*-PSSNa nanoparticles is illustrated in Figure 5. SiO_2_-NH_2_ as an inorganic compound exhibits excellent thermal stability. Its residue rate was 92.42%. The slight weight loss was probably caused by the degradation of organic functional groups on the SiO_2_ surface. SiO_2_-*g*-PSSNa and PSSNa show a similar thermal decomposition process. Nevertheless, the char residue rate of SiO_2_-*g*-PSSNa was 54.57%, which was higher than that of PSSNa. This is due to the fact that the structure of SiO_2_-*g*-PSSNa contains SiO_2_ core with higher thermal stability. Therefore, TGA measurements provided further evidence that SiO_2_-*g*-PSSNa was obtained as an organic/inorganic hybrid graft polymer. Moreover, in our study, PSSNa was not completely degraded at 800 °C. This result may be attributed to the high thermal stability of the benzene ring; while PSSNa significantly degrades at high temperatures, it does not completely degrade [43].

### 3.3. Influence of the Dosage of SiO_2_-g-PSSNa on the Apparent Viscosity of CWSs

**Figure 6:**
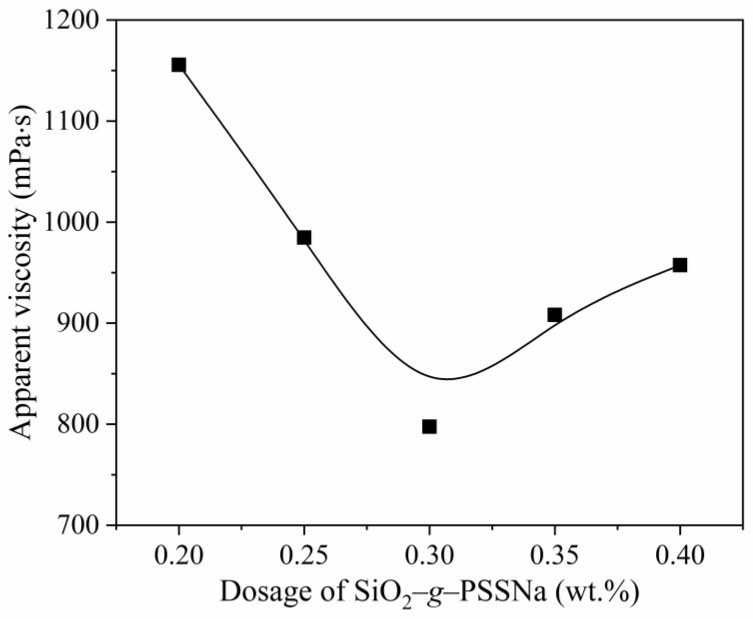
Apparent viscosity of CWSs prepared with different dosages of SiO_2_-*g*-PSSNa dispersant.

Figure 6 displays the impact of SiO_2_-*g*-PSSNa dosage on the apparent viscosity of CWSs. Evidently, with the increase in SiO_2_-*g*-PSSNa dosage, the apparent viscosity of CWS initially decreased and then increased. As we know, the electrostatic repulsion and steric hindrance between coal particles are the key factors affecting the viscosity of CWS. As the SiO_2_-*g*-PSSNa dosage increased, more SiO_2_-*g*-PSSNa molecules were adsorbed onto the coal surface, which grants coal particles enhanced electrostatic repulsion and steric hindrance force. Accordingly, the apparent viscosity of CWS was decreased. Nevertheless, when the adsorption of SiO_2_-*g*-PSSNa reached saturation, the excess SiO_2_-*g*-PSSNa was dispersed in the CWS system freely. The counterions (Na^+^) located at the PSSNa segments of dispersed SiO_2_-*g*-PSSNa compressed the electric double layer around the absorbed coal particles, which caused a weakening of electrostatic repulsion [44]. Therefore, when the dosage of SiO_2_-*g*-PSSNa exceeded a certain value, the viscosity of CWS increased conversely. Figure 6 signifies that 0.3% is the optimal dosage.

### 3.4. Rheological Behavior of CWS Prepared Using SiO_2_-g-PSSNa as the Dispersant

**Figure 7:**
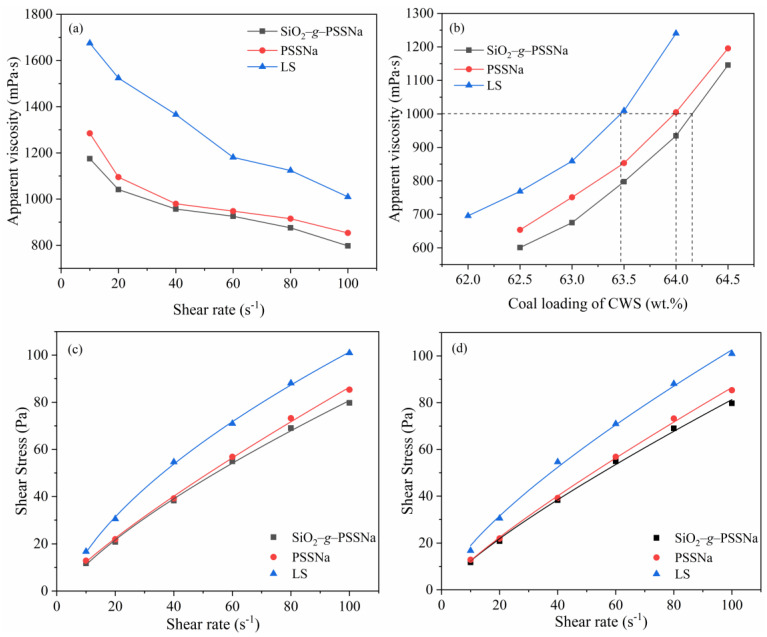
The influence of (**a**) shear rate and (**b**) coal loading on the apparent viscosity of CWSs prepared using different dispersants. Shear rate–shear stress curves fitted to (**c**) Herschel–Bulkley and (**d**) power–law models.

Rheological behavior including both apparent viscosity and fluid type has a critical influence on the practical application of CWS. Figure 7a shows the rheological curve of CWS prepared using SiO_2_-*g*-PSSNa as the dispersant. In order to fully understand the performance of this novel water-insoluble dispersant, two water-soluble commercially available products LS and PSSNa were used for comparison. In Figure 7a, three curves display similar patterns of change; with the increase in shear rate, the apparent viscosity decreased gradually. At a shear rate of 100 s^−1^, the apparent viscosity of CWS prepared with SiO_2_-*g*-PSSNa was 798 mPa·s, which was significantly lower than CWSs obtained from PSSNa (853 mPa·s) and LS (1009 mPa·s). Interestingly, SiO_2_-*g*-PSSNa and PSSNa possess approximately functional segments, whereas SiO_2_-*g*-PSSNa exhibits better viscosity-reducing performance, although SiO_2_-*g*-PSSNa is not soluble in water. We infer that this is probably due to SiO_2_-*g*-PSSNa possessing a branched structure, which was demonstrated to have a higher charge density as compared to linear structures [45,46]. When SiO_2_-*g*-PSSNa molecules are adsorbed onto the coal surface, they can grant coal particles with more electronegativity. Accordingly, the electrostatic repulsive between coal particles was enhanced, and the apparent viscosity of CWS was lowered. The shear stress–shear rate curves were fitted using Herschel–Bulkley (Figure 7c) and power–law (Figure 7d) models. Table S2 shows the obtained rheological parameters. Apparently, the rheological behavior of all CWSs is more consistent with the Herschel–Bulkley model. The rheological indices (n) of all CWSs were less than 1, indicating a pseudoplastic fluid.

An increase in the CWS concentration generates significant economic and environmental benefits [47,48,49]. Figure 7b displays the influence of coal loading and dispersant types on the apparent viscosity of CWSs. Obviously, the three curves exhibited similar patterns of change; with the increase in coal loading, the apparent viscosity of the slurry increased gradually. This is reasonable since with the increase in the coal concentration, the relative content of water in the CWS system was lowered. As a result, the apparent viscosity was increased. From the perspective of industrial application, the apparent viscosity generally should be lower than 1000 mPa·s. Therefore, Figure 7b suggests that when LS, PSSNa, and SiO_2_-*g*-PSSNa were used as the dispersants, the maximum coal loadings of CWS were 63.4%, 63.9% and 64.2%, respectively. In conclusion, Figure 7 demonstrates that failure to dissolve in water does not decrease the dispersant’s performance. SiO_2_-*g*-PSSNa exhibits an improved viscosity-reducing performance as compared to PSSNa and LS.

### 3.5. The Stability of CWSs

**Figure 8:**
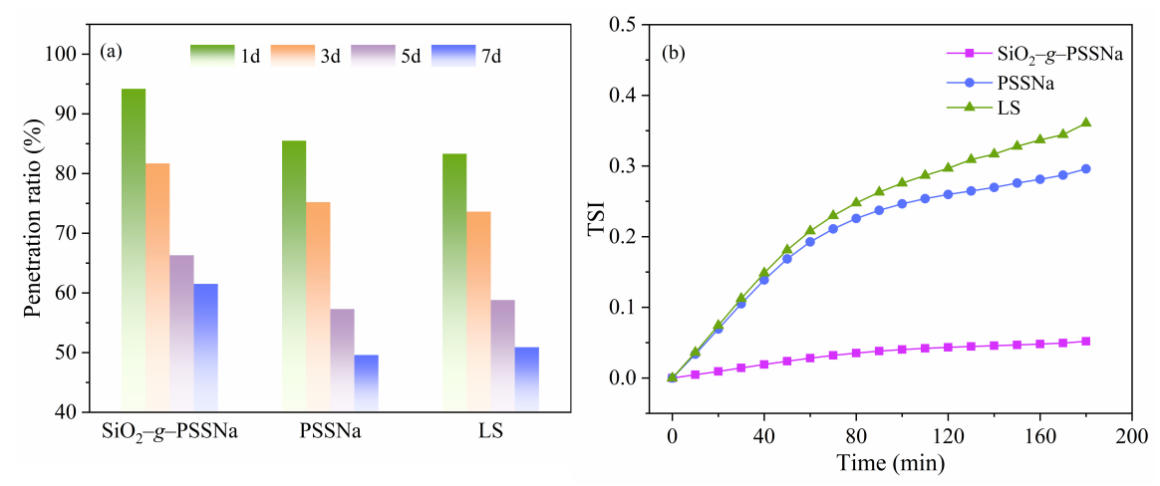
The (**a**) penetration ratio and (**b**) TSI of CWSs prepared using different dispersants.

The static stability of CWSs prepared using different dispersants was evaluated, as displayed in Figure 8. In order to ensure the reliability of the result, both penetration ratio and TSI were determined. We can conclude from Figure 8a that CWSs prepared using PSSNa and LS as dispersants exhibit approximately static stability. However, when SiO_2_-*g*-PSSNa was used as the dispersant, CWS showed an improved stability over all time intervals. Figure 8b confirmed the conclusion obtained in rod penetration tests. In Figure 8b, the TSI value of CWS prepared using SiO_2_-*g*-PSSNa was 0.05, which is significantly lower than that using PSSNa (TSI = 0.30) and LS (TSI = 0.36) as dispersants. This beneficial result is probably due to the fact that SiO_2_-*g*-PSSNa synthesized in our study is present as a water-insoluble particle on the coal surface. These particles can enhance the static repulsion between the coal particles. Thus, the aggregation of the coal particles was inhibited. Just as reported in Hu’s study, inorganic nanoparticles can be used as a stabilizer for CWS [50].

### 3.6. Dispersion Mechanism of SiO_2_-g-PSSNa

According to the established theory, several factors affect the dispersion of coal particles in the CWS system. These factors include the adsorption of dispersants, the electrostatic repulsion and steric hindrance between coal particles, water states, etc. Herein, in order to fully understand the dispersion mechanism of this novel water-insoluble dispersant, the following microscopic studies were conducted.

#### 3.6.1. Contact Angle and EDS Mapping Between SiO_2_-*g*-PSSNa Dispersion and Coal Surface

**Figure 9:**
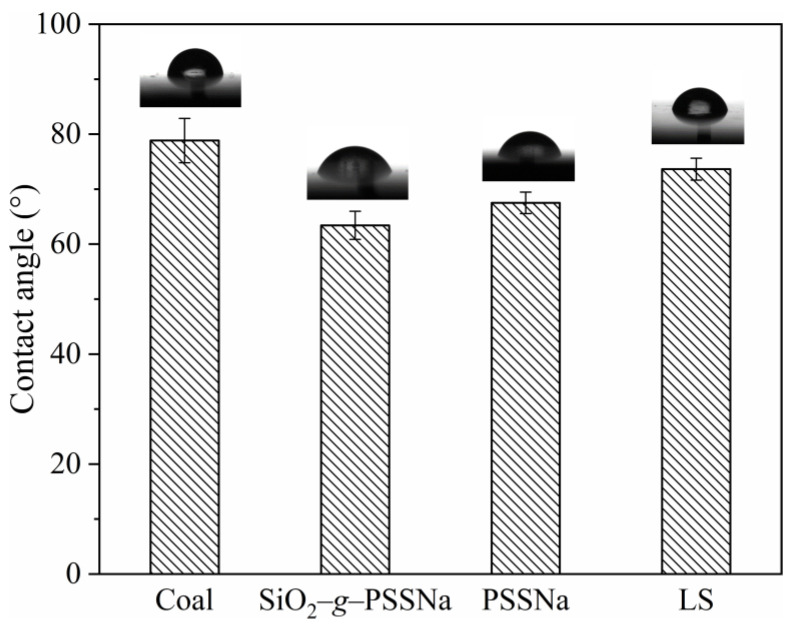
Contact angles of dispersant solutions on coal surfaces.

**Figure 10:**
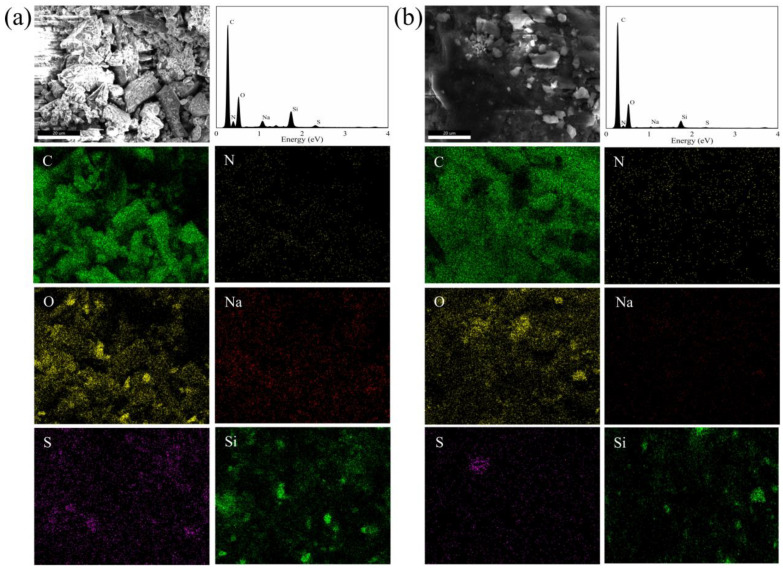
EDS mapping of (**a**) SiO_2_-*g*-PSSNa adsorbed coal particles and (**b**) raw coal.

Figure 9 shows the contact angles between dispersant solutions (or suspensions) and coal surface. The contact angles of dispersant solutions (or suspensions) were all lower than plain water, revealing that they exhibit wettability onto the coal surface. It is interesting that the SiO_2_-*g*-PSSNa suspension shows the lowest contact angle. This result implies that as conventional water-soluble polymer dispersants, SiO_2_-*g*-PSSNa can also absorb onto the coal surface. The adsorption of SiO_2_-*g*-PSSNa can also be confirmed by EDS. The raw coal and absorbed coal were subject to EDS measurements, respectively. In Figure 10a, the signals of Na, S, and Si elements on an absorbed coal surface arising from SiO_2_-*g*-PSSNa molecules were all enhanced as compared to raw coal (in Figure 10b). The quantitative results are shown in Table S3, where the content of Na, S, and Si elements were significantly higher than those of raw coal. Therefore, EDS further evidenced that SiO_2_-*g*-PSSNa is capable of adsorbing on the coal surfaces.

#### 3.6.2. Zeta Potential of Adsorbed Coal Particles

**Figure 11:**
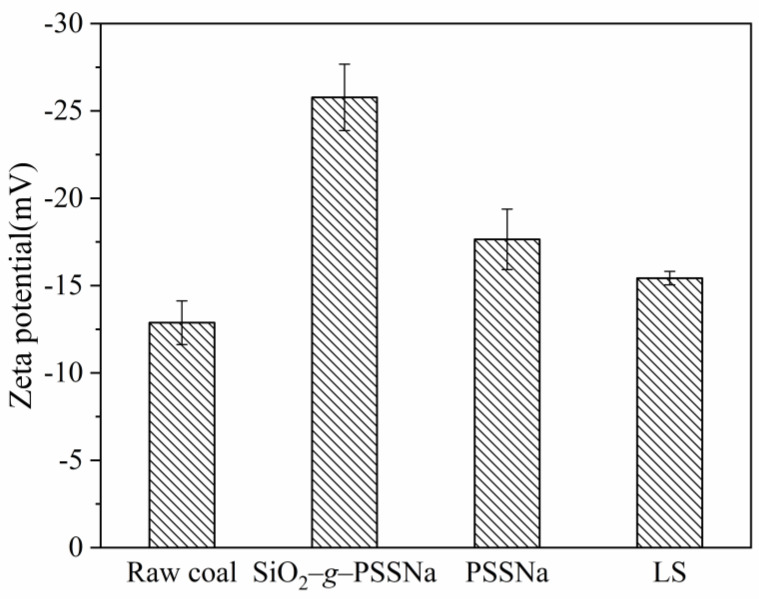
Zeta potentials of adsorbed coal particles in the CWS system.

To date, anionic dispersants have dominated industrial applications and research areas [51]. According to the eDLVO theory, electric repulsion is a crucial force for the dispersion and stabilization of coal particles. Generally, the surface of the coal is negatively charged due to the presence of oxygen-containing groups. Figure 11 suggests the zeta potentials of the coal surface increased after the adsorption of dispersants. Coal particles absorbed with SiO_2_-*g*-PSSNa exhibit enhanced electronegativity. This result validates our inference in Section 3.4 that due to the intrinsic properties of the branched-chain compounds, the chemical structure of PSSNa consists of both hydrophobic aromatic groups and hydrophilic sodium sulfonate groups. In the CWS system, the hydrophobic attraction between the aromatic ring in the PSSNa segment and thick rings on the coal surface induces the adsorption of SiO_2_-*g*-PSSNa. At this time, the ionic groups in SiO_2_-*g*-PSSNa were oriented towards water, which provides enhanced electronegativity to the coal surface [31]. Of course, the steric potential resistance between the coal particles and dispersants should also be considered. But overall, hydrophobicity attraction plays a major role. It has been proven that the hydrophobic interaction energy is 2–3 orders of magnitude higher than electrostatic interaction energy [13,52]. The enhancement of electrostatic repulsion between coal particles inhibits the aggregation of coal particles. Overall, the coal particles are dispersed under the combined action of electrostatic repulsion and steric resistance.

#### 3.6.3. Water States in CWS Systems

**Figure 12:**
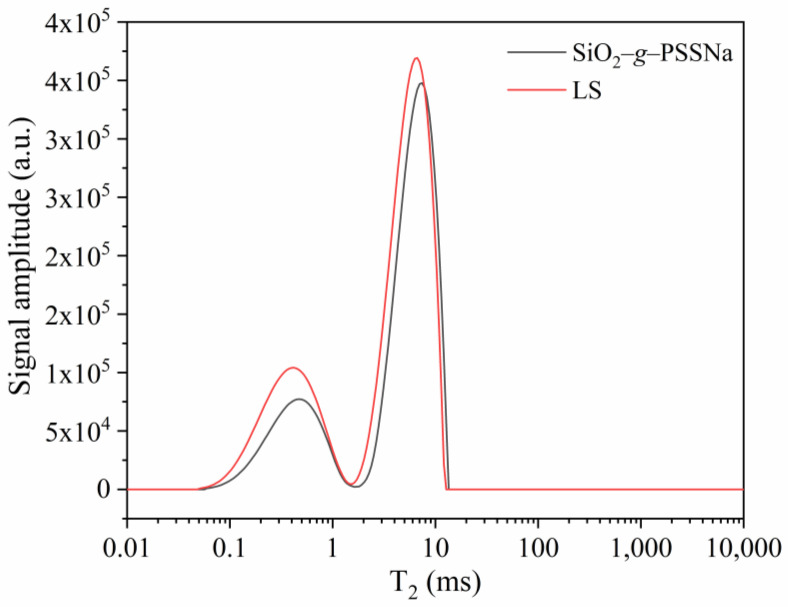
T_2_ spectra of CWSs prepared using LS and SiO_2_-*g*-PSSNa as dispersants, respectively.

Low-field NMR was employed to identify the water states in CWSs prepared using LS and SiO_2_-*g*-PSSNa as dispersants, respectively [45,53]. The T_2_ spectra are depicted in Figure 12, and the quantitative results are listed in Table 3. In general, the aggregation of coal particles will cause some free water to be restricted between the particles, and the proportion of free water will decline accordingly. Figure 12 and Table 3 indicate that the free water content in CWS prepared using SiO_2_-*g*-PSSNa as a dispersant is higher than that of LS. This result was further confirmed when SiO_2_-*g*-PSSNa was used as a dispersant, the aggregation behavior of the coal particles was seriously inhibited and more free water was released.

**Figure 13:**
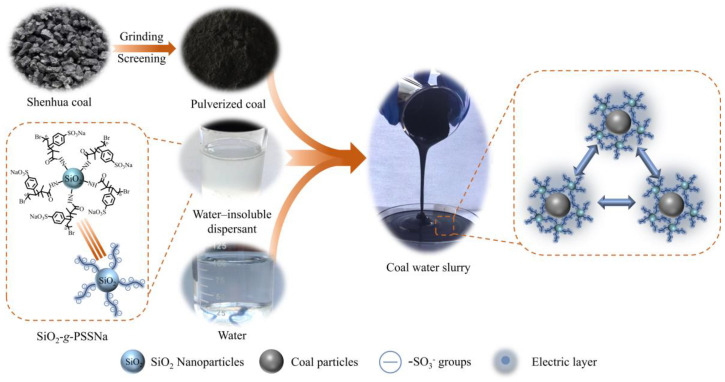
The dispersion mechanism of SiO_2_-*g*-PSSNa.

In conclusion, the dispersion mechanism of SiO_2_-*g*-PSSNa is illustrated in Figure 13. Different from conventional water-soluble polymer dispersants, this study provides a novel water-insoluble dispersant. This dispersant is composed of a SiO_2_ core and PSSNa side segments. It is dispersed in water rather than dissolved. When used as CWS dispersant, it is capable of adsorption through the interaction between the aromatic rings in side segments and on the coal surface. Due to the inherent features of branched compounds, such as a high density of functional groups and spatial size. When SiO_2_-*g*-PSSNa is aggregated and adsorbed onto the coal surface, it can grant a stronger electronegativity to the coal particles than PSSNa and LS with a linear structure. The aggregation behavior of the coal particles was inhibited by intense electrostatic repulsion. As a result, more free water was released. Therefore, apparent viscosity was lowered. As the aggregation of coal particles was suppressed, the static stability of the CWS was also enhanced.

## 4. Conclusions

A novel water-insoluble dispersant composed of a SiO_2_ core and PSSNa side segments was synthesized via the combination of surface acylation reaction and SI-ATRP, namely, SiO_2_-*g*-PSSNa. The chemical structure of SiO_2_-*g*-PSSNa was verified by various technologies, including FTIR, XPS, TGA, EDS, NMR, and Zeta potential measurements. SiO_2_-*g*-PSSNa can be used as a powerful dispersant for CWS. Its optimal dosage is 0.3%. In this situation, CWS prepared with SiO_2_-*g*-PSSNa as a dispersant exhibits lower apparent viscosity and excellent stability as compared to that obtained from PSSNa and LS, which are famous commercially available products. The coal loading can rise to 64.2% when SiO_2_-*g*-PSSNa is used as the dispersant. Low-field NMR, SEM-EDS, contact angles and Zeta potential measurements were employed to elucidate the dispersion mechanism of SiO_2_-*g*-PSSNa. Similar to conventional water-soluble polymer dispersants, SiO_2_-*g*-PSSNa can be aggregated and absorbed onto the surface of coal particles. Due to SiO_2_-*g*-PSSNa bearing a higher charge density as compared to linear PSSNa and LS. Coal particles absorbed with SiO_2_-*g*-PSSNa exhibit higher electronegativity. Stronger electrostatic repulsion enables CWS prepared using SiO_2_-*g*-PSSNa to have lower viscosity and better stability. This study confirms that water-insoluble particles with a large number of charged groups can also be used as dispersants for CWS.

## Figures and Tables

**Table 1:** The elemental composition of SiO_2_-*g*-PSSNa determined by XPS and EDS, respectively.

Testing Technology	Samples	C (%)	O (%)	Si (%)	N (%)	Br (%)	Na (%)	S (%)
XPS	SiO_2_-NH_2_	33.61	47.62	18.53	0.24	-	-	-
	SiO_2_-Br	46.64	39.55	13.29	0.36	0.16	-	-
	SiO_2_-*g*-PSSNa	65.35	24.00	1.31	0.29	0.09	4.52	4.44
SEM-EDS	SiO_2_-NH_2_	30.89	8.57	58.20	2.34	-	-	-
	SiO_2_-Br	26.33	16.63	53.94	2.60	0.50	-	-
	SiO_2_-*g*-PSSNa	44.09	16.74	32.19	1.94	0.12	2.66	2.26

**Table 2:** Zeta potentials of SiO_2_-NH_2_ and SiO_2_-*g*-PSSNa nanoparticles.

Samples	SiO_2_-NH_2_	SiO_2_-*g*-PSSNa
Zeta potential (mV)	2.12 ± 0.9	−23.53 ± 1.69

**Table 3:** The water state in CWS prepared using SiO_2_-*g*-PSSNa and LS as dispersants.

Dispersants	Content of Bound Water (%)	Content of Free Water (%)
LS	29.78	70.22
SiO_2_-*g*-PSSNa	24.77	75.23

## Data Availability

The data presented in this study are available on request from the corresponding author.

## Supplementary Materials

The following supporting information can be downloaded at: https://www.mdpi.com/article/10.3390/polym17010021/s1, Figure S1: SEM images (a) and size distribution (b) of SiO_2_-NH_2_; Figure S2: SEM (a) and TEM (b) images of SiO_2_-*g*-PSSNa; Table S1: Proximate and ultimate analysises of Shenhua coal used in this work; Figure S3: The particle size distribution of the coal sample used in this work; Figure S4: Aqueous solutions of (a) SiO_2_-NH_2_ and (b) SiO_2_-*g*-PSSNa that stand for a week; Table S2: Rheological parameters calculated based on two rheological models; Table S3: The elemental composition of raw coal and SiO_2_-*g*-PSSNa adsorbed coal particles.
