# Supplementary material for: Poly(sodium styrene sulfonate)-Grafted SiO2 Nanoparticle: Synthesis and Use as a Water-Insoluble Dispersant for Coal Water Slurry
Source: Polymers (Basel). 2024 Dec 25;17(1):21. doi: 10.3390/polym17010021 (PMC11722859; doi:10.3390/polym17010021)
Supplement: Supplementary file 1 [file polymers-17-00021-s001.zip › polymers-3362797-supplementary.pdf]

Poly(sodium styrene sulfonate)-grafted-SiO_2 nanoparticle: Synthesis and used as a water-insoluble dispersant for coal water slurry

Guanghua Zhang [a,b,*], Ruijun Liu [b], Wanbin Zhang [a,c,*], Kangmin Zhang [b], Junfeng Zhu [b], Ce Zhang [a,b]

[a] *Shaanxi Collaborative Innovation Center of Industrial Auxiliary Chemistry and Technology, Shaanxi University of Science and Technology, Xi'an 710021, China*

[b] *Shaanxi Key Laboratory of Chemical Additives for Industry, College of Chemistry and Chemical Engineering, Shaanxi University of Science and Technology, Xi'an 710021, China*

[c] *Key Laboratory of Auxiliary Chemistry and Technology for Chemical Industry, Ministry of Education, Shaanxi University of Science and Technology, Xi'an 710021, China.*

[*] *Corresponding authors, E-mail addresses: zhanggh@sust.edu.cn (G. Zhang), zhangwanbin@sust.edu.cn (W. Zhang).*

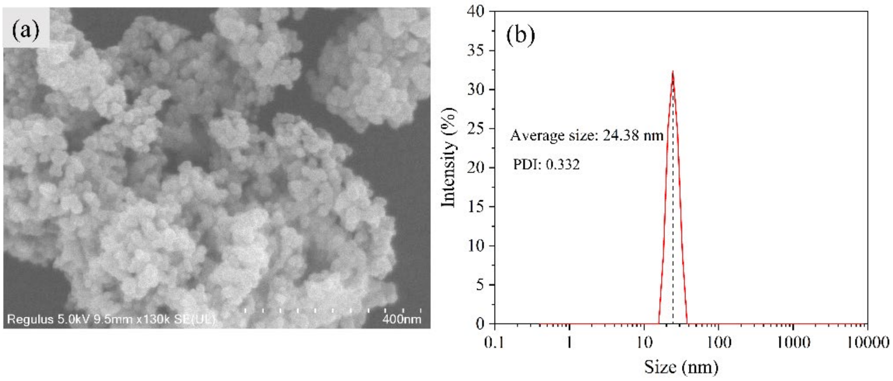

Figure S1. SEM images (a) and size distribution (b) of SiO_2-NH_2

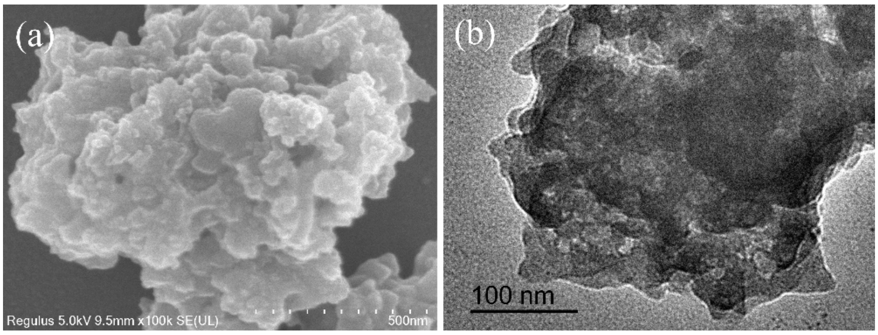

Figure S2. SEM (a) and TEM (b) images of SiO_2-g-PSSNa

Table S1 Proximate and ultimate analysises of Shenhua coal used in this work.

Proximate analysis(wt.%)				Ultimate analysis(wt.%)				
M_{ad}	V_{ad}	A_{ad}	FC_{ad}	C_{ad}	H_{ad}	O_{ad}	N_{ad}	$S_{t,ad}$
11.33	28.20	6.67	53.07	68.01	3.85	9.06	0.84	0.24

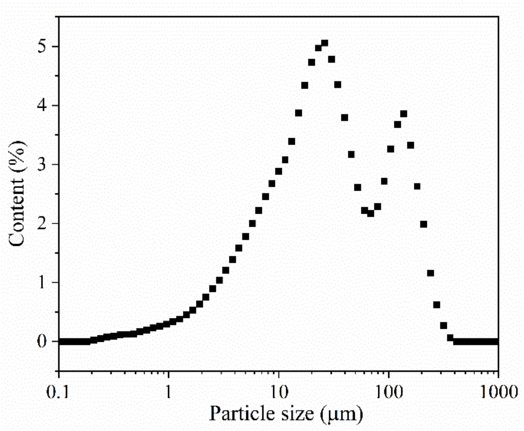

Figure S3. The particle size distribution of the coal sample used in this work.

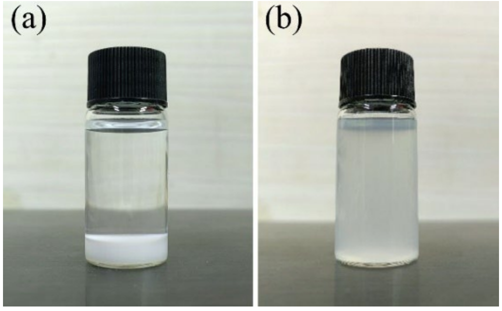

Figure S4. Aqueous solutions of (a) SiO_2-NH_2 and (b) SiO_2-*g*-PSSNa that stand for a week.

Table S2 Rheological parameters calculated based on two rheological models.

Dispersants	Herschel-Bulkley			Power-law		
	$k/(Pa\cdot s^n)$	n	R^2	$k/(Pa\cdot s^n)$	n	R^2
SiO_2-PSSNa	3.0078	0.7273	0.9979	1.8808	0.8179	0.9974
PSSNa	1.9281	0.8272	0.9985	1.8107	0.8395	0.9980
LS	7.9219	0.5812	0.9991	3.4927	0.7340	0.9965

Table S3 The elemental composition of raw coal and SiO_2-*g*-PSSNa adsorbed coal particles.

Samples	C (%)	O (%)	Si (%)	N (%)	Na (%)	S (%)
SiO_2-*g*-PSSNa adsorbed coal	69.64	24.20	1.77	3.63	0.47	0.69
Raw coal	71.88	23.26	0.91	3.67	0.17	0.10
